# Adaptive Digital Problem-Solving Skills for Life Management in the Middle Age

**DOI:** 10.1093/geroni/igaf122.2406

**Published:** 2025-12-31

**Authors:** Takashi Yamashita, Donnette Narine, Giyeon Kim

**Affiliations:** University of Maryland, Baltimore County, Baltimore, Maryland, United States; University of Maryland, Baltimore County, Baltimore, Maryland, United States; Chung-Ang University, Seoul, Republic of Korea

## Abstract

Life management digital tasks, such as paying bills, checking health information, and navigating appointments online, are essential for one’s life. In today’s fast-changing society, adults must adapt and solve problems quickly using constantly evolving digital technologies. However, middle-aged and older adults tend to be more digitally divided than their younger counterparts. Practice engagement theory depicts that continuous skill use is crucial for maintaining and enhancing one’s skills over the life course. Establishing the skill use habit in earlier adult life stages may prevent being digitally left behind in later life. Therefore, the current study examined the associations between the proficiency and utilization of adaptive digital problem-solving (ADPS) skills in middle-aged adults. We used a nationally representative sample (50 years and older; n = 1,203) drawn from the 2023 U.S. Program for the International Assessment of Adult Competencies (PIAAC) data. PIAAC provides ADPS digital skill assessment data based on the simulation of everyday life situations (e.g., planning a grocery shopping and business meeting using the digital map). The ADPS skill proficiency score (0-500 points) was regressed on the ADPS skill use and individual characteristics (e.g., age, sex, education, employment). Results showed that middle-aged adults who used ADPS skills for life management at least once a week had significantly greater ADPS skill proficiency (b = 15.98, p < 0.01) than their counterparts. Engagement with ADPS skills in middle age has implications for individual autonomy in later life in digital-technology-rich societies. Theoretical explanations and policy implications were discussed.

